# Psychological mechanisms and interventions directed at vaccination attitudes

**DOI:** 10.3389/fsoc.2023.1076015

**Published:** 2023-02-08

**Authors:** Sidonie Ann, Oliver Baumann

**Affiliations:** School of Psychology, Bond University, Gold Coast, QLD, Australia

**Keywords:** vaccine, COVID, attitudes, health, anti-vaccination

## Abstract

Attitudes about vaccination impact not only the individual but also society. Therefore, understanding the underlying psychological processes of those who disagree with vaccination is critical for creating compassion through understanding and change through promoting autonomy. The current review aimed to fill a gap in the literature, outlining the state of the recent research on vaccination attitudes, specifically on the underlying mechanisms driving anti-vaccination movements and individuals' thoughts and behaviors. In addition, we aimed to evaluate current research on the effectiveness of interventions targeting these mechanisms. Overall, results indicated that those declining vaccines had beliefs related to distrust in the scientific community and pharmaceutical companies and moral preferences for purity and liberty. In addition, our review identified the potential for utilizing motivational interviewing techniques as an intervention. This literature review provides a platform for further research and enhances the current understanding of vaccination attitudes.

## Introduction

Vaccines are widely considered the product of modern medicine preventing infectious diseases from remaining prevalent in society (Hodson, 2019). The modern scientific pursuit of vaccines is first documented in the 1700s (Riedel, 2005). Government-initiated mass vaccination started in 1840 with the United Kingdom's Vaccination Act (Wolfe and Sharp, 2002). Since then, the implementation of vaccines has resulted in deadly diseases, such as measles and whooping cough, becoming rare and a complete worldwide eradication of smallpox (Asturias et al., 2016; National Center for Immunization and Respiratory Diseases, 2020). Implementation of vaccines facilitated not only the immunization of the vaccinated individual but also that of those around them. Herd immunity is a biological and physical phenomenon in society for which a threshold level of individuals has been immunized, facilitating a significant decrease in the disease transmission rate (Berezin and Eads, 2016). This indicates that individual vaccination is a personal choice of societal concern on a global scale. It should be noted, however, that herd immunity is sometimes very difficult to attain, due to waning immunity, or pathogen mutability, and it is unobtainable for diseases that are not spread from person to person, including tetanus (Fine, 1993).

Vaccines are a preventative measure usually administered to healthy individuals and, as such, are held to a high standard of safety (Asturias et al., 2016). However, the widespread use of vaccines has led to concerns over their safety in recent decades (Asturias et al., 2016). A potential risk of immunization is a vaccine's side effect profile, an effect produced in the body separate from the vaccine's intended effect (Health and Human Services, 2021). Common side effects of most vaccines are usually mild and go away without intervention and can include pain or swelling at the injection site, mild fever, chills, fatigue, headache, muscle or joint aches, and fainting (Health and Human Services, 2021). While these side effects are unpleasant, they indicate that the body's immune system is beginning to recognize and build a defense against that disease (Health and Human Services, 2021). A severe side effect is anaphylaxis, which is, however, extremely rare. The U.S. Department of Health and Human Services (2021) reports the incidences of severe allergic reactions to be 1–2 people per million doses.

In addition to the side effects, the literature reports that in rare cases, adverse events following immunization (AEFI) occur largely unrelated to the vaccine itself (Asturias et al., 2016; Okuhara et al., 2020). Asturias et al. (2016) acknowledge that the misunderstanding about these reactions and false literature has led to the misattribution of side effects and AEFI and subsequent negative public opinions.

### Anti-vaccination movement

There are numerous broad definitions in the literature on vaccine hesitancy and refusal, depending on the nature of their investigation into the underlying mechanisms of the behavior. However, for this review, vaccine hesitancy is defined as a delay in acceptance or refusal of vaccines, despite the availability of vaccination services (Peretti-Watel et al., 2015). There are those who are hesitant about vaccines but still partake in the immunizations; however, there are also those who are actively objecting to the use of vaccines and subsequently do not partake in immunizations (Salmon et al., 2015; Asturias et al., 2016; Bocquier et al., 2018; Wiley et al., 2020). The latter are categorized as refusers and are the focus of the current review (Salmon et al., 2015; Asturias et al., 2016).

With the scientific discovery of vaccines came the politics of government decision-making, and with it, counter-movements, known as anti-vaccination activists (Nour, 2019). The motive behind these movements was to fight against the compulsory nature of vaccinations holding the view that this was a violation of individual liberties rather than opposition to vaccines themselves (Nour, 2019).

The health belief model (HBM) is a theoretical model employed to guide health promotion and disease prevention programs (Nour, 2019). It is used to explain and predict individual changes in health behaviors, and it argues that “the two components of health-related behavior are (1) the desire to avoid illness, or conversely get well if already ill; and, (2) the belief that a specific health action will prevent, or cure, illness” (Boston University School of Public Health, 2022, “The Health Belief Model” section). The model illustrates the risk–benefit analysis in that an individual would analyze the danger of developing the sickness and the value of behaving to offset this risk (Nour, 2019). When applied to vaccines, it describes and predicts how people evaluate the risk of susceptibility to a disease that a vaccine protects against the danger connected with the sickness and the hazards associated with the vaccine (Nour, 2019). Aligned with the HBM are the theory of planned behavior (TPB) and the social cognitive theory (SCT) (Nour, 2019). The TPB outlines that intentions and perceived control influence decisions to engage in a particular behavior. The SCT proposes that goals, outcome expectancies, self-efficacy, and sociostructural variables influence an individual's behavior (Nour, 2019). It is essential to investigate these variables, as these are where interventions could be targeted.

### Strategies addressing vaccine attitudes

Anderson et al. (2020) highlight that the current strategies provided to physicians by the Center for Disease Control in the U.S. facilitating vaccine-refusing families' informed decision-making are an empathic approach. However, the downsides of this approach are the time it takes to convey empathy effectively and build trust, which is an expensive resource in clinical settings. Furthermore, having vaccine-resistant individuals in clinical settings exposes vulnerable patients and staff to vaccine-preventable diseases (Anderson et al., 2020).

Much of the literature addressing vaccine refusal includes removing barriers, such as access to vaccines and affordability (Anderson et al., 2020). Some governments have been guided by this research, such as Australia's implementation of pharmacists administering vaccines to increase access (National Centre for Immunisation Research and Surveillance, 2021). This targets an environmental factor identified as a barrier to access; however, this approach does not address the underlying psychological mechanisms. In their systematic review of strategies to address parents' refusal of childhood vaccinations, Nour (2019) identified mass marketing and direct communication as two strategies proposed in the literature. Mass marketing aimed to counteract the misinformation spreading, while direct communication was in line with the CDC recommendations that healthcare professionals build a relationship of trust (Nour, 2019).

This review aims to identify the underlying psychological mechanisms of individuals against vaccinations and the interventions designed to target those to improve vaccination uptake. It is important to note that the review of interventions is not aimed to override individual autonomy but rather balance autonomy with unhelpful psychological underpinnings and facilitate a more thorough understanding and compassion (Rozbroj et al., 2019b). There is currently a gap in the literature outlining the state of the research to date and the success of current interventions. Specific research questions for this review include.

- What underlying mechanisms drive vaccination-refusing movements and individual thoughts and behaviors?
- What are the current interventions targeting these mechanisms, and how effective are they?

## Method

### Search strategy

A systematic search, selection, and review of the existing literature were conducted concerning vaccination-refusing typologies and intervention strategies. The current review was guided by, and the results are written following the Preferred Reporting Items for Systematic Reviews and Meta-Analyses (PRISMA; Page et al., 2021). Relevant articles from the following databases were searched: Cochrane, PubMed, PsychInfo (Ovid), and ProQuest between May and September 2021. The following terms were utilized, individually and in combination, in the search: “anti-vaxxers,” “anti-vaccination,” “vaccine refusal,” “vaccine hesitancy,” “psychological determinants AND anti-vaxxers,” “personality traits AND anti-vaxxers,” “immunization theories AND vaccine refusal,” “increasing vaccination OR vaccine uptake,” “vaccine literacy,” “anti-vaxxer AND interventions,” “interventions vaccine hesitancy AND vaccine refusal,” “vaccine refusal AND anxiety OR fear,” “vaccine refusal OR hesitancy AND attitudes OR beliefs,” and “vaccine refusal AND health anxiety.” It is important to note that while hesitancy was not the focus of the study, the literature is mixed on the definition of hesitancy and refusal; therefore, to encapsulate the research as a whole, hesitancy was included as a search term and the exclusion criteria were applied at the study selection step. The searches were limited to English texts and peer-reviewed, published articles only. Subsequently, reference lists were also searched for relevant records.

### Study selection and data extraction

#### Study selection

Following PRISMA, the Cochrane “PICO” method was utilized to refine eligibility criteria for the inclusion of studies in the current review (Page et al., 2021; McKenzie et al., 2022). PICO defines criteria surrounding population, intervention, comparator, and outcome for studies investigating interventions (McKenzie et al., 2022). The scope of these parameters can be broad or narrow depending on the research question (McKenzie et al., 2022). In the present review, the population parameter included individuals with anti-vaccination beliefs or behaviors. The intervention parameter was broad, given the explanatory nature of our review, and included all empirical-design studies. Similarly, no parameters were set for the comparator or outcome parameters, which is a common approach for exploratory reviews (McKenzie et al., 2022). Furthermore, no limitations were applied to age, geographical location, or type of vaccination.

The PRISMA protocol recommends additional parameters regarding research design, language, and publication status (Page et al., 2021). The design parameter included specific measures or questions identifying psychological underpinnings of anti-vaccination beliefs or behaviors, excluding studies with a limited focus on prevalence and sociodemographic factors. As stated previously, the search was limited to English text only and peer-reviewed published articles. Subsequently, titles and abstracts were evaluated for relevance, regarding the target population (i.e., individuals with anti-vaccination beliefs or behaviors) and design (i.e., empirical studies), and duplicates were removed. Subsequently, the remaining records underwent full-text screening and were subjected to a research quality appraisal conducted following the Mixed Methods Appraisal Tool (MMAT) (Hong et al., 2018).

**Figure 1:**
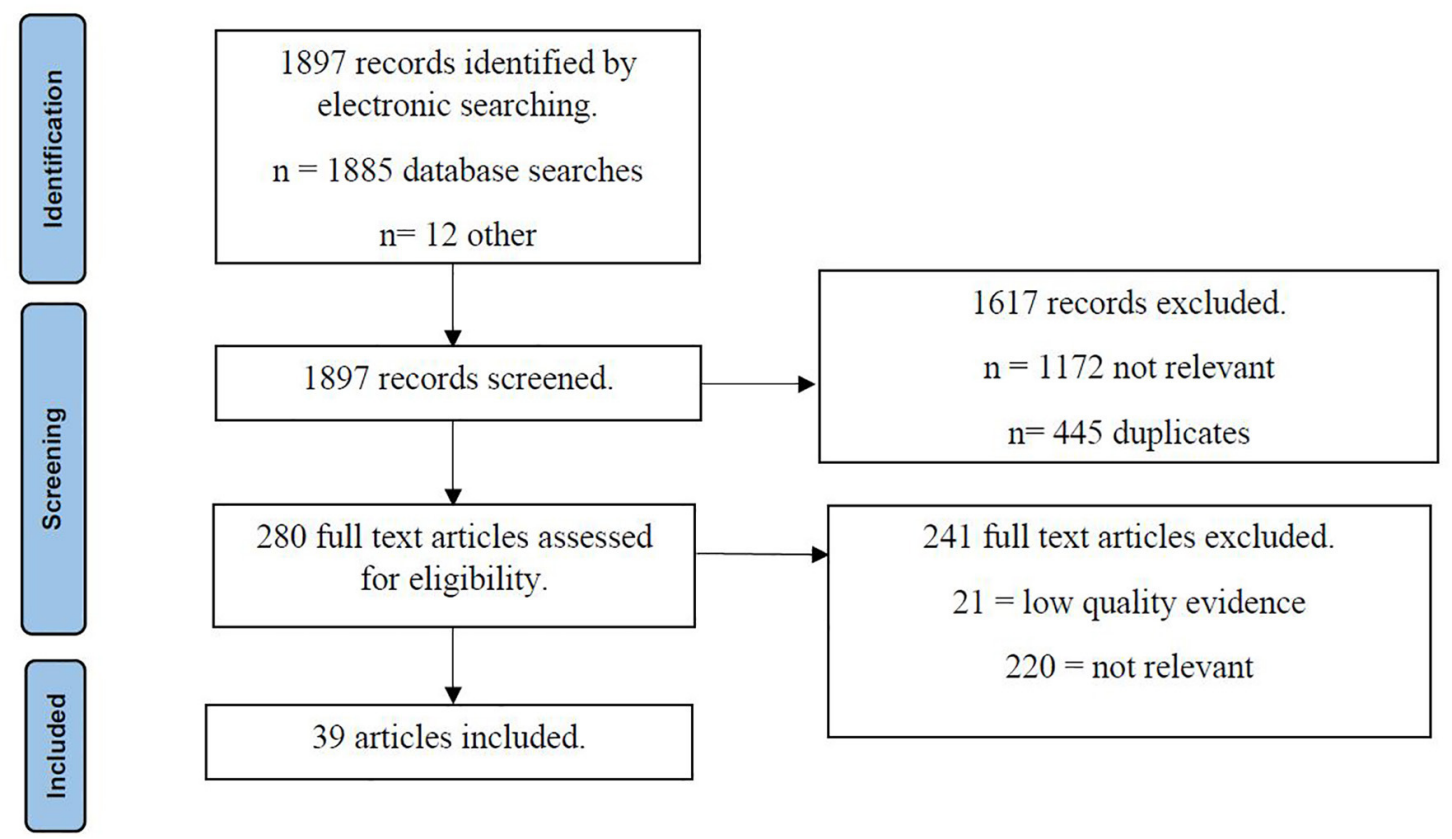
Study selection.

The MMAT is a tool designed to facilitate the appraisal of research for review studies based on methodological quality (Hong et al., 2018). First, studies were excluded if the research question was unclear, or the data did not address the research question. Second, studies were examined regarding the appropriateness of data collection methods and measures, representativeness of samples, risk of bias, and appropriateness of the statistical analysis. Specifically, studies were deemed low quality and excluded if they were not relevant to the scope of the study, did not represent the target population (e.g., focus on hesitancy without refusal), focused solely on barriers to vaccination (e.g., access to vaccinations), were theoretical only, did not define data collection methods (e.g., no specific measures/questions identifying anti-vaccination attitudes), or were statistically underpowered (i.e., with very small sample sizes or effect size). Figure 1 demonstrates the selection process.

#### Data extraction

The extracted data included the year of publication, authors, population demographics, methodology, variables, outcomes, strengths, and limitations (including the quality of the study).

## Results

### Study selection

A total of 1,897 records were retrieved *via* the search strategy outlined previously. Subsequent title and abstract screening resulted in 1,617 records being excluded from the present review. From the remaining 280 records, 241 were excluded after a full-text screening, with exclusion criteria concerning study quality (utilizing the MMAT), relevance to anti-vaccination, underlying mechanisms, or interventions. Therefore, a total of 39 studies were included in the current review.

### Study characteristics

All studies included in the review were conducted between 2004 and 2021. The number of studies investigating the underlying mechanisms of anti-vaccination attitudes and beliefs was 33 (Smith et al., 2004; Fowler et al., 2006; Luyten et al., 2014; Michael et al., 2014; Wada and Smith, 2015; Reich, 2016; Amin et al., 2017; Chung et al., 2017; Bryden et al., 2018; Carrion, 2018; Hornsey et al., 2018; Motta et al., 2018, 2021; Restivo et al., 2018; Tustin et al., 2018; Bianco et al., 2019; Carpiano et al., 2019; Helps et al., 2019; Romijnders et al., 2019; Rossen et al., 2019; Rozbroj et al., 2019a, 2020, 2022; Cadeddu et al., 2020; Goldberg and Richey, 2020; Tomljenovic et al., 2020; Dzieciolowska et al., 2021; Elkalmi et al., 2021; Freeman et al., 2021; Huynh and Senger, 2021; Martinez-Berman et al., 2021; Murphy et al., 2021). Within this subset of studies, 12 studies investigated parents as a sample (Smith et al., 2004; Reich, 2016; Amin et al., 2017; Chung et al., 2017; Carrion, 2018; Tustin et al., 2018; Bianco et al., 2019; Carpiano et al., 2019; Helps et al., 2019; Romijnders et al., 2019; Rossen et al., 2019; Tomljenovic et al., 2020), 20 adults as a sample (Fowler et al., 2006; Luyten et al., 2014; Michael et al., 2014; Wada and Smith, 2015; Bryden et al., 2018; Hornsey et al., 2018; Motta et al., 2018, 2021; Restivo et al., 2018; Rozbroj et al., 2019a, 2020, 2022; Cadeddu et al., 2020; Goldberg and Richey, 2020; Dzieciolowska et al., 2021; Elkalmi et al., 2021; Freeman et al., 2021; Huynh and Senger, 2021; Murphy et al., 2021), and one was web based (Martinez-Berman et al., 2021).

The number of studies investigating the efficacy of interventions targeting this population was six (Dempsey et al., 2015; Pot et al., 2017; Gagneur et al., 2018; Lemaitre et al., 2019; Pluviano et al., 2019; Nowak et al., 2020). Of this, five were investigating parents of children who require vaccinations (Dempsey et al., 2015; Pot et al., 2017; Gagneur et al., 2018; Lemaitre et al., 2019; Pluviano et al., 2019), and one focused on the general adult population (Nowak et al., 2020).

Of all studies, eight utilized empirically validated measures (Amin et al., 2017; Bryden et al., 2018; Hornsey et al., 2018; Bianco et al., 2019; Freeman et al., 2021; Huynh and Senger, 2021; Martinez-Berman et al., 2021; Murphy et al., 2021), while the remaining studies utilized semi-structured interviews, surveys, and questionnaires designed specifically for the study, and web-based data mining.

Table 1 displays the characteristics of the studies included, including aims, study design, intervention/measures, outcomes, and strengths and limitations.

**Table 1:** Study characteristics.

**References**	**Aim**	**Participants**	**Design**	**Intervention, measures, and/or vaccination**	**Outcome**	**Strengths and limitations**
Amin et al. (2017)	To investigate the underlying moral values of individuals who are vaccine hesitant (categorized into low, medium or high hesitancy; with high hesitancy reflective of refusal)	Study 1: *N* = 1,007 parents. U.S. based aged 18–50, and parent of minimum 1 child under 13 years Study 2: *N* = 464. U.S based on MTurk, parent of minimum 1 child, 18 years or over Random sample	Two correlational cross-sectional online surveys	Parent attitudes about childhood vaccines (short scale), the moral foundations questionnaire, and liberty foundation questionnaire	Results indicated that purity and liberty foundations are highly correlated with highly vaccine hesitant parents and low advocacy of authority	Strengths: correlational study, use of validated measures, high statistical power overall. Anti-vaccination clearly defined Limitation: unable to infer causality, possible low generalisability due to inclusion of low and medium hesitancy
Bianco et al. (2019)	The aim was to investigate attitudes toward childhood vaccines among parents	Italy. *N* = 385. Parents of kindergarten children (1–5 years) Purposive sample	Cross-sectional survey	Parent attitudes about childhood vaccines (PACV) to measure vaccine hesitancy; questionnaire involving questions socio-demographic information, vaccine experiences, beliefs and attitudes about vaccination, risk/benefit, trust in the health system and pharmaceutical industry, media exposure, influential leaders, and politics	Vaccine refusing parents: more likely to view childhood vaccines as an economic business of pharmaceuticals and who disagree with mandates that only vaccinated children should be allowed to attend kindergarten	Strengths: use of validated measures, high statistical power with focus on vaccine refusal Limitation: unable to infer causality
Bryden et al. (2018)	To investigate whether an individual's general health worldview might explain a relationship between complementary and alternate medicine (CAM) and vaccine skepticism	*N* = 2,697 Australian adults aged 18–89 Convenience sampling	Cross-sectional survey involving individuals from an institutional health survey panel	International questionnaire to measure use of complementary and alternative medicine (CAM measurement), measurement formulated for the present study examining vaccine skepticism, and the general magical beliefs subscale of the magical beliefs about food and health scale	CAM and anti-vaccination were related in terms of attitudes for which an individual's health worldview accounted for a significant proportion. Therefore, vaccine skepticism attitudes relating to general health discounting general scientific knowledge in favor of superstitious thinking	Strengths: correlational study, large sample size Limitation: no use of validated tool to identify anti-vaccination attitudes. Unable to infer causality
Cadeddu et al. (2020)	To investigate the socio-cultural profile of Italians based on their vaccination beliefs	*N* = 2, 626 Italy. 15 + years Random probability sampling	Data was collected from the European Social survey (face-to-face interviews)	Principal component analysis (PCA) and latent class analysis (LCA)	Those holding anti-vaccination beliefs tended to distrust the scientific community, lower levels of engagement in political and cultural life, and tended to be male and of older age	Strengths: large sample size, use of PCA and LCA, interview facilitating depth of understanding Limitation: no use of validated tool to identify anti-vaccination attitudes, however, LCA utilized in assisting to overcome this, Unable to infer causality, possible social desirability bias impacting more than surveys
Carpiano et al. (2019)	To investigate the association of socioeconomic status with knowledge, attitudes and beliefs (KAB) of vaccines; and actual vaccination behavior	Canadian. *N* = 24, 853 parents of children ages 2, 7, 12–14, and 17 (immunization ages for measles, mumps, and rubella vaccination) Purposive sampling	Cross-sectional survey from 2013 childhood national immunization coverage survey	Survey investigated information regarding the parent's KAB, as well as parent and child demographics. KAB was investigated utilizing two variables; lack of vaccine confidence and potential side effects	Results indicated that there were SES differences in KAB, which focused on vaccine side effects and subsequent safety. Two to seven year immunisations negatively-oriented KAB evidenced in parents with post-secondary education levels (non-university graduates) and lower to upper middle class incomes. Twelve to fourteen KAB limited to safety in disadvantaged and middle-class households	Strengths: large sample size Limitations: did not capture those who refused all vaccines, however, this facilitated comparison to hesitancy. Unable to infer causality
Carrion (2018)	To explore the underlying reasons and decision-making about parental vaccine refusal	U.S. based. *N* = 50. 21–41-year-old mothers Purposive sampling	Cross-sectional semi-structured interview	Open-ended questions about explanations for vaccine decision-making process and reasoning, inclusive of when/why first refusal	Results suggested that vaccine refusal was a result of three health considerations; perceived adverse reactions, endorsements from health care professionals against vaccination, and perceived inconsistency among expert-endorsed messages	Strengths: sample focussed on vaccine refusal. Interviews facilitate deeper understanding Limitations: no use of validated tool to identify anti-vaccination attitudes. Unable to infer causality. Possible social desirability bias impacting more than surveys. Small sample size—limited generalisability
Chung et al. (2017)	To explore influences on parental decision-making regarding vaccines	U.S based. Two surveys (2012 *N* = 2,603; 2,014 *N* = 2,518). Parents of children under 7 years Probability proportional to size sampling	Cross-sectional web-based surveys	Initial screening questions relating to vaccine decision-making. Follow-up questions relating to sources of vaccine advice and information, influences of vaccine decision-making, social networks of refusal	Vaccine refusers select their healthcare provider based on whether they would facilitate their intention not to vaccinate. Additionally, they are more likely to know someone whose child experienced an adverse reaction	Strengths: large sample size, across two time point to capture trends, sampling improved generalisability Limitations: no use of validated tool to identify anti-vaccination attitudes. Unable to infer causality
Dempsey et al. (2015)	To investigate the efficacy of an iPad- based intervention on vaccines by the impact on vaccine attitudes and behavior	*N* = 42. Parents in three primary care clinics. U.S based Purposive sampling	Pre- and post-surveys assessing vaccination intentions and attitudes. Medical records were utilized to examine actual uptake	Tailored messaging “TeenVaxScene” information presented to parents of adolescents	Along with their finding that the tailored message had little significant impact on attitudes and behavior, they also found that parents were highly unlikely to engage with this particular format	Strengths: pre and post-test design, as well as reviewing medical records measure subsequent behavior Limitations: no use of validated tool to identify anti-vaccination. Small sample size, with smaller sample of refusers limiting generalizability. No control group
Dzieciolowska et al. (2021)	To determine levels of COVID-19 vaccine acceptance among healthcare workers, and underlying reasons for hesitancy	*N* = 2,761 nurses, healthcare managers, environmental services workers and physicians from a Canadian multicentre institution	Cross-sectional survey. Surveys were administered *via* emails regarding the healthcare workers eligibility for COVID-19 vaccinations	Original surveys were designed by the investigators and included questions regarding how important factors were in their decision	When compared with other healthcare workers (physicians, healthcare managers, etc.) nurses were the least likely group to accept the vaccine. Vaccine refusers were more likely to have a distrust for pharmaceutical companies and preference for development of natural immunity	Strengths: large sample size, including large sample of vaccine refusers improving generalisability Limitations: no use of validated tool to identify anti-vaccination attitudes, unable to infer causality
Elkalmi et al. (2021)	To explore the attitudes, religious beliefs, and familiarity regarding vaccination in religious and science students	Malaysia. *N* = 300 students of religious studies and applied sciences (pharmacy) Convenience sampling	Cross-sectional survey	38 item questionnaire—reasons for not supporting vaccines, attitudes toward vaccination, religious beliefs toward vaccination, familiarity with vaccinations, and religious activities	Results indicated that those who did not support vaccine included religious reasons and harm associated with the vaccine	Strengths: large sample size. Novel variable of religion Limitations: no use of validated tool to identify anti-vaccination. Small sample of refusers. Limited generalisability due to sampling method, unable to infer causality
Fowler et al. (2006)	To explore the factors influencing individuals' decision to vaccinate (anthrax vaccine)	U.S. based. *N* = 404 from 44 Laboratory Response Network laboratories, limited to laboratory technicians, laboratory supervisors, environmental investigators, and other laboratory employees deemed high risk to exposure Purposive sampling	Cross-sectional survey	Decision-making survey inclusive of demographic information, perceived risk of exposure, safety concerns of vaccine, access to information facilitating an informed decision, most important factor underlying vaccine decision, and credibility of the vaccine information statement	Those who refused the vaccines were more likely to rate their risk of exposure to anthrax as low, note concerns surrounding vaccine safety, and highlight a distrust of the vaccine safety statement	Strengths: large sample size. Sampling method limits generalisability but highlights a situation that risk is able to be quantified in a workplace (novel design) Limitations: no use of validated tool to identify anti-vaccination attitudes, unable to infer causality
Freeman et al. (2021)	To evaluate the proportion of vaccine hesitant individuals who have blood-injection-injury fears	U.K. based. *N* = 14, 149. Adults 18+ Non-probability sampling	Cross-sectional online survey assessing intention to be vaccinated against COVID-19, as well as survey investigating injections fears	Screening question included vaccine refusal The Oxford COVID-19 vaccine hesitancy scale, specific phobia scale- blood-injection-injury phobia, and medical fear survey-injections and blood	Blood-injection-injury phobia explained a small to medium proportion of vaccine hesitancy and vaccine refusal	Strengths: large sample size, clear definition, appropriate design and analysis, use of validated measures Limitations: small proportion of vaccine refusal limiting generalisability, unable to infer causality
Gagneur et al. (2018)	To assess the effect of a motivational-interviewing educational strategy of vaccination promotion	Canada. Maternity Ward. Mothers (18 years +, English/ French speaking). Mothers requiring acute care were excluded. *N* = 2,483; 1,140 (experimental) and 1,343 (control) infants involved in the study Purposive sampling	Quasi-experimental. Over a period of 1 year. Delivered once to consenting mothers 24–48 h post-partum (experimental group); control group was those mothers who were “screened out” or did not consent	MI-based intervention adhering to the Quebec Immunization protocol on infant vaccine and vaccine preventable diseases	Vaccine rates and feasibility of the intervention Intervention significantly increased vaccination rate of infants	Strengths: large sample size, clear definition, appropriate design and analysis. Matched control group Limitations: no use of validated measure to identify anti-vaccination attitudes
Goldberg and Richey (2020)	To examine the underlying mechanism of anti-vaccination beliefs; specifically, how they related to belief in conspiracies	*N* = 4,230 U.S. based from the ANES 2016 pre/post-election survey Random sampling	Interviews were conducted face-to-face, and *via* a web-based platform.	Two questions regarding anti-vaccination (risk), Obama being a Muslim, trutherism (did the government know about the 9/11 attacks), trust in government, political knowledge, and to measure authoritarianism the Social Conformity Scale	Anti-vaccination beliefs are demonstrated to be a part of a psychological propensity to believe in conspiracies	Strengths: large sample size, clear definition, appropriate design and analysis. Use of validated measure regarding authoritarianism Limitations: no use of validated measure to identify anti-vaccination attitudes
Helps et al. (2019)	To examine vaccine refusal decisions in parents.	Australia. *N* = 32 non-vaccinating parents (9 fathers and 22 mothers) Purposive sampling	Cross-sectional semi-structured interviews in a defined population of low vaccination rates	Interviews covered topics inclusive of reasons for not vaccinating (of ceasing vaccinations), how the decision was made within the family and the influences of others such as health professionals, the media and government policies	Results suggested that parents who refuse vaccinations included perceived deterioration in health in western societies, a personal experience regarding vaccine safety, concerns regarding consent, encounters with health professionals (dismissive, indexing, and helpful)	Strengths: exclusive focus on anti-vaccination improving generalisability within this population, clear definition, appropriate design and analysis Limitations: no use of validated measure to identify anti-vaccination attitudes, small sample size, unable to infer causality
Hornsey et al. (2018)	To examine the underlying psychological factors impacting on motivation to rejection scientific consensus regarding vaccination	24 nations *N* = 5,323 adults	Cross-sectional survey. Measuring antivaccination attitudes, conspiracy theory beliefs, reactance, disgust sensitivity toward blood/needles, individualistic/hierarchical worldviews	Vaccine beliefs: 7 items from the beliefs about vaccine safety and efficacy subscale of the parent attitudes about vaccines scale Individualism-hierarchy worldview: Cultural cognition worldview scale Reactance: Hong psychological reactance scale Disgust: blood and injection subscale of the disgust emotion scale Conspiratorial beliefs-questionnaire designed for the study	Results indicated that anti-vaccination attitudes were highest amongst those who demonstrated conspiratorial thinking, high reactance, high level of disgust toward blood/needles, strong individualistic/hierarchical worldviews	Strengths: large sample size, clear definition, appropriate design and analysis. Large, equal number of participants from different countries increasing generalisability, use of validated measures Limitations: measure to identify anti-vaccination was not appropriate for sample, subsequently, only a few items were utilized decreasing validity, unable to infer causality
Huynh and Senger (2021)	Investigate the relationship between intellectual humility with anti-vaccination attitudes, intention to vaccinate against COVID-19	*N* = 351 participants. USA based, 18+ Random sampling	Cross-sectional online survey	Comprehensive intellectual humility scale (intellectual humility), vaccine attitudes examination (VAX) scale (anti-vaccination attitudes), adaptation of the flu vaccine intention scale (vaccination intentions)	Negative association between anti-vaccination attitudes and intellectual humility	Strengths: large sample size, clear definitions, appropriate design and analysis, including power analysis Limitations: small subset of sample held anti-vaccination beliefs limiting generalisability, unable to infer causality
Lemaitre et al. (2019)	To assess the effect of an educational strategy of vaccination promotion based on MI techniques on long-term vaccination uptake	Canada. Maternity Ward. Mothers (18 years +, English/French speaking). Mothers requiring acute care were excluded. *N* = 2,483; 1,140 (experimental) and 1,343 (control) infants involved in the study Purposive sampling	Quasi-experimental. Over a period of 1 year. Delivered once to consenting mothers 24–48 h post-partum (experimental group); control group was those mothers who were “screened out” or did not consent	Experimental group included an individual education session with motivational interviewing techniques (PromoVac)	The experimental group had a higher chance at the child completing a vaccination schedule	Strengths: large sample size appropriate design and analysis Limitations: no use of validated measure to identify anti-vaccination—utilized vaccination status. No definition related to anti-vaccination. Therefore, hard to generalize to those individuals who hold anti-vaccination view specifically. No use of validated measures
Luyten et al. (2014)	To explore the differences in psychological disposition, specifically societal orientation, between vaccine skeptics and non-skeptics	Belgium. *N* = 1,050. Adults, 18+ age Random sampling	Cross-sectional survey	Triandis and Glefand Social Orientation Scale and one question regarding attitude toward vaccines “if a vaccine exists for a certain disease, then vaccination is usually a good way to protect someone against the disease”	Vaccines skeptics have a different social orientation, specifically, vaccine skeptics are lower on horizontal individualism and horizontal collectivism indicative of a lower propensity to view others as equals	Strengths: large sample size, including large sample of vaccine refusers improving generalisability, appropriate design and analysis Limitations: no use of validated measure to identify anti-vaccination attitudes, unable to infer causality
Martinez-Berman et al. (2021)	To determine if there is a relationship between anti-vaccination attitudes and interest in, or admiration for, celebrities	*N* = 320. Adults, U.S based Random sampling	Online cross-sectional survey	Celebrity attitude scale and vaccination attitudes examination scale	Significant positive association between anti-vaccination attitudes and celebrity admiration. No correlation between celebrity admiration and lack of trust in vaccine safety	Strengths: large sample size, focus on anti-vaccination attitudes, clear definition, appropriate design and analysis, use of validated measures Limitations: unable to infer causality
Michael et al. (2014)	To explore the underlying reasons for polio vaccine refusal	Nigeria. *N* = 148. LGA with a history of continued OPC refusals Purposive sampling	Cross-sectional semi-structured interviews	Semi-structured interview regarding perceived polio threat, reasons for OPV refusal, perceptions of campaigns and vaccination teams	Results indicated that perception risk of polio was low, additionally, vaccine refusal was impacted by views that the vaccine was not necessary or helpful., the vaccine may be harmful, and religious beliefs (e.g., The power of God)	Strengths” sample of vaccine refusers improving generalisability, appropriate design and analysis, clear definition Limitations: no use of validated measure to identify anti-vaccination attitudes, small sample, unable to infer causality
Motta et al. (2018)	To evaluate if the Dunning-Kruger effect can explain anti-vaccination attitudes	International based study. *N* = 1,310 adults Random sampling	Cross-sectional online survey	Survey questions on topics relevant to health policy, knowledge of autism, overconfidence, anti-vaccination attitudes, group decision-making	Overconfidence (belief in knowing more than doctors and scientists about autism) is associated with increased support for non-experts (e.g., celebrities) and opposition to mandatory vaccination policy	Strengths: Large sample size, diverse sample with specific focus on vaccine refusers improving generalisability, appropriate design and analysis. Clear definition Limitations: no use of validated measure to identify anti-vaccination attitudes, unable to infer causality
Motta et al. (2021)	To investigate the social identity of individuals that that identify as anti-vaccination	*N* = 1,001 U.S. based adult population Random sampling	Cross-sectional online survey	Survey to identify vaccine attitudes- then administered a measure of their social identity (novel measure)	Those who identify as anti-vaccination individuals embrace it as a form of social identity, they also have a tendency toward distrust in scientific experts and are more individualistic	Strength: large sample size, with specific focus on vaccine refusers improving generalisability, appropriate design and analysis. Clear definition Limitations: no use of validated measure to identify anti-vaccination attitudes, unable to infer causality
Murphy et al. (2021)	To understand COVID-19 vaccine hesitancy and resistance in terms of psychological mechanisms and sociodemographic factors	Ireland (*N* = 1,041) and the United Kingdom (*N* = 2,025). Adult population Random sampling	Cross-sectional survey	Measures included: The Big Five Inventory, locus of control, The Cognitive Reflection Task, The Identification with all Humanity Scale, The Conspiracy Mentality Scale, the Persecution and Deservedness Scale, Monotheist and Atheist Beliefs Scale, a question on participants trust in government, British Social Attitudes Scale, The Very Short Authoritarianism Scale, the Social Dominance Scale	Results indicated that those resistant to a COVID-19 vaccine were less likely to engage information from traditional and authoritative sources and had lower levels of mistrust in these sources. Additionally, more self-interested, religious beliefs, conspiratorial and paranoid beliefs. Further, they were more disagreeable, impulsive, emotionally unstable and less conscientious	Strength: large sample size, with specific focus on vaccine refusers improving generalisability, appropriate design and analysis. Clear definition Limitations: no use of validated measure to identify anti-vaccination attitudes, unable to infer causality
Nowak et al. (2020)	To investigate the impact of supplementing vaccine information statements with messages of community immunity benefits of influenza vaccination in a virtual reality format would improve vaccine-avoidant perceptions, beliefs, confidence, and intentions	U.S. Based. *N* = 171 participants aged 18–49 vaccine avoidant Purposive sampling	One-way between-subjects experimental design	Virtual reality, short video, pamphlet, and control group	Results indicated that the VR group had the most effect on increased concern about transmitting influenza, increased positive vaccine related beliefs and subsequently associations with higher intention to vaccinate	Strengths: appropriate sample size overall, appropriate design and analysis, use of control group, clear definition Limitations: No use of validated measure to identify anti-vaccination, small sample of anti-vaccination therefore hard to prove generalisability
Pluviano et al. (2019)	To determine whether the “Myth vs. facts” pro-vaccination strategy is an effective tool to counter vaccine misinformation	Italy. *N* = 60 parents attending pediatricians' surgeries Purposive Sampling	Pre- and post-test design with participants randomly allocated to each experimental and control group	Preliminary questionnaire involved questions regarding participant's beliefs and attitudes toward vaccines. The same survey was administered after the intervention and then again 7 days later	The intervention resulted in stronger vaccine misconceptions. Therefore, this is not an effective intervention	Strengths: small sample size, appropriate design and analysis—use of control group, clear definition Limitations: no use of validated measure to identify anti-vaccination attitudes, limited generalisability
Pot et al. (2017)	To evaluate the effectiveness of a vaccine promoting web-based intervention	*N* = 8,062 Dutch mothers recruited *via* Dutch vaccination register Purposive sampling	RCT. Participants were assigned to the control group, or the intervention group. Surveys were administered pre and post, as well as the vaccination register to determine vaccine uptake	Computer-based tailored intervention	No effects were found on HPV vaccination uptake	Strengths: large sample size overall, small sample of anti-vaccination participants, appropriate excellent design and analysis—use of control group and RCT, clear definition Limitations: no use of validated measure to identify anti-vaccination, small sample of anti-vaccination therefore hard to prove generalisability
Reich (2016)	To examine the underlying reasons parents, refuse vaccines for their children	U.S. parents, pediatricians and complementary health providers who oppose vaccines. *N* = 34 parent interviews (29 were mothers) Purposive sampling	Cross-sectional interview and observation	Semi-structured interviews and ethnographic observation at national conferences that oppose vaccines, analysis of parenting forums	Parents view their infants as “naturally perfect” lacking need of protection, vaccines are perceived as artificial, unnatural and dangerous with a preference for natural immunity as belief it is superior. their perceptions of immunity include natural is superior	Strengths: Focus on anti-vaccination participants, appropriate design and analysis-qual deeper understanding, clear definition Limitations: no use of validated measure to identify anti-vaccination attitudes, small sample therefore hard to prove generalisability
Restivo et al. (2018)	To investigate the factors associated with refusal of the HPV vaccine among young women	Italy. *N* = 141 women 18–25 years, who had at least 1 vaccination among all included in the Sicilian vaccination schedule who have not started or completed HPV vaccination. 84% of participants were unvaccinated,15% had one dose of HPV Purposive sampling	Cross-sectional telephone survey	Questionnaire, based on the Health Belief Model framework, included 23 items on HPV infection and vaccination knowledge	Results indicated that refusal of HPV was associated with a bachelor's level education, lower participation at school seminar on HPV, and lower perception of HPV vaccine benefits	Strengths: adequate sample size, focus on anti-vaccination behavior, appropriate design and analysis Limitations: no use of validated measure to identify anti-vaccination attitudes, unclear definition, unable to infer causality
Romijnders et al. (2019)	To investigate the underlying factors involved in parents' decision-making about childhood vaccination	U.S. based. *N* = 197 (19 acceptors, 12 refusers, and 24 partial acceptors) parents of children Purposive sampling	Cross-sectional semi-structured focus groups, grouped by acceptors, refusers, and partial acceptors	Interviews included questions relating to knowledge, attitudes, decision-making processes and information needs relating to vaccination	Refusers and partial acceptors reported increased deliberation in decision-making process compared with acceptors, with answers indicating that their knowledge was occasionally lacking scientific evidence, and perceived risk of VPDs low, while risk of adverse reaction high, lower trust in welfare centers and vaccine information provided to them	Strengths: adequate sample size, appropriate design and analysis—including grouping individuals with similar beliefs to reduce social desirability bias, clear definition Limitations: no use of validated measure to identify anti-vaccination attitudes, unable to infer causality, small sample of anti-vaccination individuals
Rossen et al. (2019)	To determine the underlying moral roots of individuals with anti-vaccination attitudes	Australia. *N* = 296 Parents or guardians targeted *via* parenting websites Purposive sampling	All participants were administered 3 questionnaires assessing their attitudes toward vaccination, behavioral intentions and moral preferences (novel measure created for vaccine confidence)	Latent profile analysis to characterize each group (acceptors, rejectors, fence sitters)	Rejectors of vaccines exhibited an increased moral preference for liberty (rights of the individual), harm (concern about the wellbeing of others), purity (abhorrence for impurity of the body), and decreased moral preference for authority (deference to those in power positions)	Strengths: adequate sample size, appropriate design and analysis, clear definition Limitations: no use of validated measure to identify anti-vaccination attitudes, unable to infer causality
Rozbroj et al. (2019a)	To examine the psychosocial characteristics relating to health and government that underly attitudes toward childhood vaccines.	Australia. *N* = 4,370 Adults aged 18+ Random sampling.	Cross-sectional survey-the Australian vaccine survey	Survey measured vaccine attitude and psychosocial attributes relating to healthcare, health consumerism, and government	Compare with their positive counterparts, those with negative attitudes toward childhood vaccines were more informed, engaged and independent health consumers, demonstrated greater adherence to complementary medicine, high distrust in “mainstream” healthcare system, higher conspiratorial thinking and more likely to align with minor political parties	Strengths: large sample size overall, appropriate design and analysis, clearly defined anti-vaccination Limitations: no use of validated measure to identify anti-vaccination attitudes, unable to infer causality
Rozbroj et al. (2020)	To investigate the extent to which having children influenced parent's vaccine beliefs (specifically, vaccine hesitant and vaccine-refusing parents)	Australia. *N* = 904 Parents aged 18+ Purposive sampling.	Cross-sectional survey	The survey included questions regarding their beliefs toward vaccine and whether their beliefs changed after having children	Onset of parenthood prompted individuals to learn about vaccines, hesitant and refusing parents interpreted this information with a distrust of pharmaceutical companies and regulatory bodies	Strengths: adequate sample size, focus on anti-vaccination behavior, appropriate design and analysis, clear definition Limitations: no use of validated measure to identify anti-vaccination attitudes, unable to infer causality
Rozbroj et al. (2022)	To examine how individuals who identify with the anti-vaccination movement described the movement and its meaning	Australia. *N* = 696. Adults aged 18+ Purposive sampling	Cross-sectional, online survey	Survey questions examined attitudes toward vaccination	Beliefs included: a distrust for vaccine promotion and supporting scientific evidence, view themselves as well-informed and science-based with goal of promoting scientific values and advocating for better vaccine research	Strengths: large sample size, focus on anti-vaccination behavior, appropriate design and analysis, clear definition Limitations: no use of validated measure to identify anti-vaccination attitudes, unable to infer causality
Smith et al. (2004)	To examine the characteristics of children who have no vaccines, compared with those who are under-vaccinated	U.S. Based. *N* = 21 163. Parents of children aged 19–35 months who had 1 or under recommended vaccines Representative probability	Cross-sectional survey and interviews.	Stage 1: interview demographic, socioeconomic, vaccine history and vaccine decision-making Stage 2: Vaccination histories obtained from medical providers	Under-vaccinated children tend to have a younger mother lacking higher education, live near the poverty level, and live in central city, whereas unvaccinated children tended to have a united family with a mother holding higher education, higher annual income and have parents who hold concerns regarding the safety of vaccines and indicated that medical professionals have little influence in their decision-making	Strengths: large sample size, focus on anti-vaccination behavior, appropriate design and analysis, clear definition Limitations: no use of validated measure to identify anti-vaccination attitudes
Tomljenovic et al. (2020)	To explore the factors involved in vaccine conspiracy beliefs and vaccine uptake in children	Croatia. *N* = 823. Parents Purposive sampling	Cross-sectional survey	Parenting survey, not vaccine-specific topics. Rational-experiential Inventory, Life-Orientation Test-revised, Vaccine Conspiracy Beliefs Scale, and the Emotions toward Vaccination Scale	Greater vaccine conspiracy beliefs were associated with stronger unpleasant emotions toward vaccines, greater experientially intuitive thinking, and lower education levels. Vaccine refusal was also associated with unpleasant emotions toward vaccination and intuitive thinking	Strength: large sample size, focus on anti-vaccination behavior, appropriate design and analysis Limitations: no use of validated measure to identify anti-vaccination, unclear definition of anti-vaccination attitudes, unable to infer causality
Tustin et al. (2018)	To investigate the association between parents perception of risk of childhood immunization with seeking vaccine information on the internet	Canadian-based sample, *N* = 966. Parents of children 0–15 years old Purposive and random sampling	Cross-sectional web-based and telephone survey	Questions surrounding primary source of information on vaccines, and a measure on perception of risk of vaccines	Parents with negative attitudes toward vaccine were more likely than their counterparts to search the internet for information on vaccines	Strengths: large sample size, focus on anti-vaccination behavior, appropriate design and analysis, clear definitions Limitations: no use of validated measure to identify anti-vaccination attitudes, unable to infer causality
Wada and Smith (2015)	To investigate the association between mistrust for government vaccine recommendations and the socio-demographic characteristics of individuals in Japan	Japan-based. *N* = 3,140 adults 20–69 years (working age) Random sampling	Web-based cross-sectional survey	Questionnaire (novel creation) related to trust in the government regarding vaccinations, the most trusted source of information about vaccines, general health	Individuals who reported mistrust in the official government sources were more likely to consider friends, the internet and books, family and newspapers (women), and television (men) as the most trusted sources for vaccination-related information. Poor health among men was associated with general mistrust of vaccination recommendations	Strengths: large sample size, focus on anti-vaccination behavior, appropriate design and analysis, clear definition Limitations: no use of validated measure to identify anti-vaccination attitudes, unable to infer causality

### Key findings

The studies investigating the underlying mechanisms of anti-vaccination attitudes and beliefs identified anti-vaccination attitudes to be related to lower levels of intellectual humility (Tomljenovic et al., 2020; Huynh and Senger, 2021) with the specific belief that the individual knows more than a medical professional (Motta et al., 2018). Nineteen studies identified beliefs associated with anti-vaccination attitudes, specifically distrust in the scientific community (Fowler et al., 2006; Carrion, 2018; Helps et al., 2019; Romijnders et al., 2019; Cadeddu et al., 2020; Motta et al., 2021), distrust in pharmaceuticals (Bianco et al., 2019; Rozbroj et al., 2019a, 2020; Dzieciolowska et al., 2021; Martinez-Berman et al., 2021), superstitious beliefs (Bryden et al., 2018), and belief in conspiracy theories (Hornsey et al., 2018; Smith and Graham, 2019; Goldberg and Richey, 2020; Tomljenovic et al., 2020; Martinez-Berman et al., 2021; Rozbroj et al., 2022). Four studies highlighted a moral profile inclusive of increased liberty (Luyten et al., 2014; Amin et al., 2017; Hornsey et al., 2018; Rossen et al., 2019); harm, purity, and decreased moral preference for those in authority (Rossen et al., 2019).

Additionally identified as being associated with anti-vaccination profiles were celebrity admiration (Martinez-Berman et al., 2021), religiosity (Michael et al., 2014; Rozbroj et al., 2019a; Elkalmi et al., 2021), reactance (Hornsey et al., 2018), belief in natural immunity and purity of the body (Michael et al., 2014; Reich, 2016), fear or disgust of blood needles (Hornsey et al., 2018; Freeman et al., 2021), belief as social identity (Motta et al., 2021), and low participation in political or cultural life (Cadeddu et al., 2020). Typical behaviors identified in the literature were that those that held anti-vaccination attitudes were more likely to search the internet or other sources of information rather than seek information from government or health professionals (Smith et al., 2004; Wada and Smith, 2015; Chung et al., 2017; Tustin et al., 2018; Murphy et al., 2021).

The studies examining specific interventions focused on motivational interviewing, tailored web-based interventions, and education (Dempsey et al., 2015; Pot et al., 2017; Gagneur et al., 2018; Lemaitre et al., 2019; Pluviano et al., 2019; Nowak et al., 2020). Two studies investigated the utility of motivational interviewing-based strategies, with both studies reporting significant increases in vaccine uptake (Gagneur et al., 2018; Lemaitre et al., 2019). Two other studies explored the benefit of interactive-/web-based tailored messaging, with both studies reporting no significant impact (Dempsey et al., 2015; Pot et al., 2017). The final two studies examined the efficacy of education-based interventions, both resulting in no significant impacts (Pluviano et al., 2019; Nowak et al., 2020).

## Discussion

The current review aimed to identify the underlying psychological mechanisms of individuals holding anti-vaccination attitudes and the interventions designed to target these mechanisms. Our review addresses a gap in the literature by increasing the understanding of individuals with anti-vaccination beliefs and behaviors. Furthermore, our findings help to clarify the roles of health professionals in assisting with vaccine decision-making that balances individual rights with societal demands. Studies were included based on the relevance (specific focus on vaccine refusal and underlying mechanisms) and quality of the research, with a total of 39 studies, 33 on underlying mechanisms, and six investigating interventions. The literature included in this review identified three core domains underlying the psychological mechanisms related to anti-vaccination attitudes, including beliefs, morals, and individual characteristics.

### Beliefs

The overarching beliefs discovered primarily included a distrust of the scientific community and pharmaceuticals, and the presence of superstitious and conspiratorial beliefs (Smith et al., 2004; Fowler et al., 2006; Wada and Smith, 2015; Chung et al., 2017; Carrion, 2018; Hornsey et al., 2018; Tustin et al., 2018; Bianco et al., 2019; Helps et al., 2019; Romijnders et al., 2019; Smith and Graham, 2019; Cadeddu et al., 2020; Tomljenovic et al., 2020; Dzieciolowska et al., 2021; Martinez-Berman et al., 2021; Motta et al., 2021; Murphy et al., 2021; Rozbroj et al., 2022). This finding is congruent with previous research exploring linguistic themes among social media users expressing anti-vaccination beliefs (Buchanan and Beckett, 2014; Faasse et al., 2016; Okuhara et al., 2017, 2018; Smith and Graham, 2019; Dhaliwal and Mannion, 2020). Returning to the health belief model, this mistrust of vaccines and science directly impacts risk-reward decision-making processes, as the information involved in the decision-making process may not reflect validated science or health information.

### Morals

Results from the literature outline a moral profile that may typically be observed in an individual holding anti-vaccination attitudes. This moral profile includes a higher preference for morals associated with liberty, harm, and purity, with decreased moral preferences for authority (Luyten et al., 2014; Amin et al., 2017; Hornsey et al., 2018; Rossen et al., 2019). Liberty underpins attitudes surrounding rights and autonomy associated with mandated vaccinations and an individual's right to personal choices (Rossen et al., 2019). This moral preference aligns with their beliefs surrounding mistrust and the findings that typical behaviors included sourcing other avenues of information, such as celebrities, and socially aligning with others who think the same (Smith et al., 2004; Wada and Smith, 2015; Chung et al., 2017; Tustin et al., 2018; Martinez-Berman et al., 2021; Motta et al., 2021; Murphy et al., 2021). In addition, a high moral value for liberty suggests that mandates surrounding vaccines may only further exacerbate their beliefs, increase the gap between science and the community, and increase the utility of motivational interviewing as an effective tool (Kriss et al., 2022). Morals related to harm are associated with heightened attention to the detriment that may be caused through vaccines, consistent with the literature that highlights cognitions related to the risk of side effects and adverse reactions (Amin et al., 2017; Carrion, 2018; Carpiano et al., 2019; Helps et al., 2019; Rossen et al., 2019; Elkalmi et al., 2021). Morals related to purity suggest that these individuals have a deep belief in natural immunity, indicating that when conducting a risk analysis in their decision-making, the benefits associated with not vaccinating appear higher than the risks of vaccination (Rossen et al., 2019). In addition, there was a decreased moral preference for authority, suggesting a lack of support when health directives are provided and mandated (Luyten et al., 2014; Rossen et al., 2019).

### Individual differences

Two main cognitive biases were identified underpinning anti-vaccination attitudes in the research presented in this review. Motta et al. (2018) found a correlation between the Dunning–Kruger effect and anti-vaccination attitudes, indicating that those who hold anti-vaccination attitudes have overconfidence in their knowledge. This is consistent with Aechtner's (2021) study, which examined anti-vaccine websites and discovered that commentators typically believe their expertise to be superior to medical specialists. This is further congruent with other research suggesting a mistrust of the scientific community, lower likelihood to obtain information from scientific sources, discounting scientific findings, and Huynh and Senger's (2021) finding of low intellectual humility (Fowler et al., 2006; Bryden et al., 2018; Bianco et al., 2019; Rozbroj et al., 2019a; Motta et al., 2021; Murphy et al., 2021). In addition, the omission bias was identified across multiple studies (Fowler et al., 2006; Luyten et al., 2014; Michael et al., 2014; Amin et al., 2017; Hornsey et al., 2018; Rossen et al., 2019). This indicates that some individuals holding anti-vaccination attitudes engage in risk-reward decision-making from the view that omission of action (not vaccinating) is less risky than engaging in action (vaccination) (Freeman et al., 2021). This bias is congruent with the moral profile inclusive of purity, highlighting a preference for natural immunity and omission of action to decrease perceived risk. Understanding an individual's cognitive style, including cognitive biases, aids in understanding their decision-making, and how to assist to improve health and wellbeing while preserving autonomy.

Interestingly, the examined research found minimal difference between individual anti-vaccination attitudes and parent anti-vaccination attitudes, with mistrust in the scientific community and fear of adverse reactions or side effects serving as overarching themes supporting both opinions (Smith et al., 2004; Fowler et al., 2006; Wada and Smith, 2015; Chung et al., 2017; Carrion, 2018; Hornsey et al., 2018; Tustin et al., 2018; Bianco et al., 2019; Helps et al., 2019; Romijnders et al., 2019; Smith and Graham, 2019; Cadeddu et al., 2020; Tomljenovic et al., 2020; Dzieciolowska et al., 2021; Martinez-Berman et al., 2021; Motta et al., 2021; Murphy et al., 2021; Rozbroj et al., 2022). Regarding the fear of reactions and side effects, parents appeared to report more experience with adverse reactions (friends, family, or narratives); however, it must be noted that there was limited analysis into personal experience in any studies within the adult individuals group.

### Interventions

The present literature highlights the promise of motivational interviewing as a technique for health professionals to decrease vaccine refusal based on individual decision-making (Gagneur et al., 2018; Lemaitre et al., 2019). Motivational interviewing increases autonomous motivation by facilitating collaboration and empathy and positioning the individual as the “expert” on their values. It has already been used successfully in the psychological field (e.g., treatment compliance) (Widder, 2017). In line with the health belief model, motivational interviewing facilitates long-term behavioral change by acting on the morals and beliefs of the individual, i.e., respecting their right to autonomy (Fall et al., 2018).

Interestingly, two studies highlighted a fear or disgust of blood needles among those who oppose vaccination (Hornsey et al., 2018; Freeman et al., 2021). This not only emphasizes an under-researched area within the study of psychological underpinnings of anti-vaccination attitudes but also presents another avenue in which psychological interventions, such as motivational interviewing, may assist individuals.

Interactive web-based messaging and education-based interventions were found to have less utility in facilitating change (Dempsey et al., 2015; Pot et al., 2017; Pluviano et al., 2019; Nowak et al., 2020). Congruent with previous findings suggesting a significant level of distrust of the scientific community and a propensity to seek out other sources of information, it is not surprising that education-based interventions do not impact individuals with anti-vaccination attitudes (Smith et al., 2004; Fowler et al., 2006; Wada and Smith, 2015; Chung et al., 2017; Carrion, 2018; Hornsey et al., 2018; Tustin et al., 2018; Bianco et al., 2019; Helps et al., 2019; Romijnders et al., 2019; Smith and Graham, 2019; Cadeddu et al., 2020; Tomljenovic et al., 2020; Dzieciolowska et al., 2021; Martinez-Berman et al., 2021; Motta et al., 2021; Murphy et al., 2021; Rozbroj et al., 2022).

## Strengths, limitations, and future research

The strengths of the current research include the diversity of vaccines and populations studied in vaccination decision-making. Furthermore, the literature included a mixture of quantitative and qualitative methods facilitating a richer understanding. Finally, the strengths of the present review consist of the exclusion of poorer quality studies, and the dual focus on underlying mechanisms and target interventions to facilitate understanding of how the two are working together (or not).

The limitations of the current research include a lack of consistency in identifying anti-vaccination attitudes or vaccine refusals, the limited use of validated tools, the size of the target populations, and the western perspective (see Table 1 for specific studies). Within the literature, definitions of “anti-vaccination” included beliefs and behaviors ranging from entirely against vaccines and refusing uptake to viewpoints amenable to change. This inconsistency increased the difficulty of conducting the review and introduced a degree of subjectivity that the use of the MMAT tool usually decreases. In addition, only a few studies utilized validated tools to identify anti-vaccination attitudes, which could impact capturing the true nature of vaccine refusal. Future research into typologies could build upon the current research utilizing psychometrically validated tools, such as Shapiro's et al. (2018) Vaccine Hesitancy Scale.

Furthermore, it is important to highlight that intervention focused on individual attitudes is only one of the many ways in which vaccination uptake can be improved. Other important factors, such as access to and affordability of vaccines (Anderson et al., 2020), go beyond the scope of the current review.

Finally, only a few studies focused on anti-vaccination attitudes on a population level, meaning that the generalizability might be low. Similarly, the western perspective of the research with predominantly United States, Canadian, United Kingdom, and Australian-based studies also affects generalizability. This bias may be due to the limitation of the current literature review, in which only studies written in English were included. Future research should investigate other populations to gain a more holistic perspective on anti-vaccination beliefs, as it is a global issue.

## Author contributions

SA and OB contributed to conception, design, and manuscript writing. All authors have read and approved the submitted version.
